# Plausibility and parameter sensitivity of micro-finite element-based joint load prediction at the proximal femur

**DOI:** 10.1007/s10237-017-0996-1

**Published:** 2017-12-30

**Authors:** Alexander Synek, Dieter H. Pahr

**Affiliations:** Institute of Lightweight Design and Structural Biomechanics, TUW, Getreidemarkt 9/BE, Vienna, Austria

**Keywords:** Micro-finite element, Inverse remodelling, Load estimation, Sensitivity, Femur

## Abstract

A micro-finite element-based method to estimate the bone loading history based on bone architecture was recently presented in the literature. However, a thorough investigation of the parameter sensitivity and plausibility of this method to predict joint loads is still missing. The goals of this study were (1) to analyse the parameter sensitivity of the joint load predictions at one proximal femur and (2) to assess the plausibility of the results by comparing load predictions of ten proximal femora to in vivo hip joint forces measured with instrumented prostheses (available from www.orthoload.com). Joint loads were predicted by optimally scaling the magnitude of four unit loads (inclined $-20^{\circ }$ to $100^{\circ }$ with respect to the vertical axis) applied to micro-finite element models created from high-resolution computed tomography scans ($30.3~\upmu$m voxel size). Parameter sensitivity analysis was performed by varying a total of nine parameters and showed that predictions of the peak load directions (range 10$^{\circ }$–$30^{\circ }$) are more robust than the predicted peak load magnitudes (range 2344.8–4689.5 N). Comparing the results of all ten femora with the in vivo loading data of ten subjects showed that peak loads are plausible both in terms of the load direction (in vivo: $18.2\pm 2.0^{\circ }$, predicted: $20.0^{\circ }$) and magnitude (in vivo: $2707.6\pm 443.3~\hbox {N}$, predicted: $3372.2\pm 597.9~\hbox {N}$). Overall, this study suggests that micro-finite element-based joint load predictions are both plausible and robust in terms of the predicted peak load direction, but predicted load magnitudes should be interpreted with caution.

## Introduction

Knowledge of physiological bone loading conditions is highly relevant for medical applications such as predicting patient specific fracture risk (Taddei et al. 2014) or the success of fracture healing (Lacroix and Prendergast 2002; Claes et al. 1998), but is also fundamental to functional interpretations of architectural differences in bones of both living and extinct species (Skinner et al. 2015; Tsegai et al. 2013; Christen et al. 2015). Although joint loads are among the largest forces acting on a bone, their quantification remains challenging both by experimental and computational means (Bergmann et al. 2016; Rikli et al. 2007; Kim et al. 2009; Garijo et al. 2014). A relatively novel approach of estimating joint loads is to make use of the bones’ ability to adapt to its mechanical environment (Fischer et al. 1995; Christen et al. 2012). Since this method relies on bone architecture alone it is potentially applicable not only to living, but also extinct species where only bone is preserved (Bona et al. 2003, 2006; Christen et al. 2015).

**Fig. 1:**
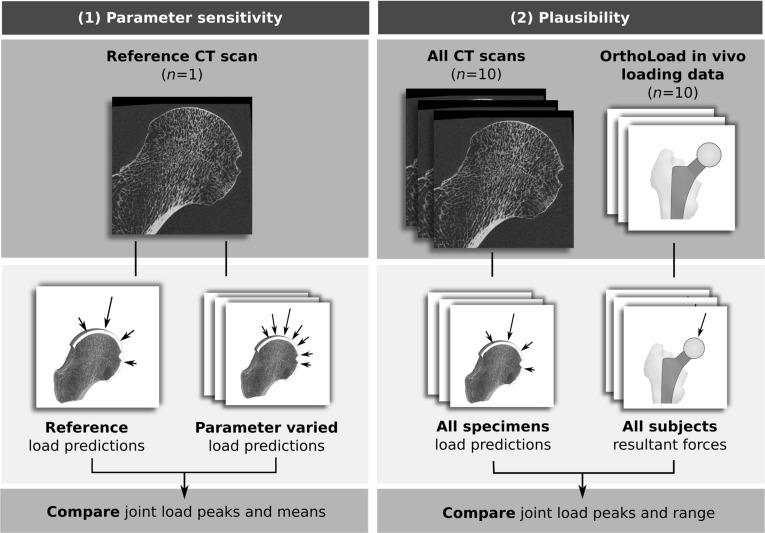
Graphical abstract of this study with two parts: (1) One specimen was selected, and predicted peak and mean joint load vectors were compared between a reference model and parameter-varied models. (2) Plausibility was assessed by comparing peak joint load vectors and ranges predicted using the micro-FE algorithm with in vivo resultant hip joint forces reported by Bergmann et al. (2016) (OrthoLoad data, www.orthoload.com)

The main idea of estimating joint loads from bone architecture is to find a set of loading conditions which leads to a state of remodelling equilibrium; a state where bone is neither added nor resorbed (Fischer et al. 1995). The bone loading estimation can be implemented efficiently by solving finite element (FE) models for a predefined set of unit loads and finding optimal load scaling factors that lead to the most homogeneous load distribution within the tissue (Fischer et al. 1995; Christen et al. 2012). Using micro-FE models, this method was successfully used to predict in vivo changes of loading conditions in mice vertebrae (Christen et al. 2012) and has been validated with forward remodelling algorithms in small bone cubes (Christen et al. 2013). In a preliminary study, it was also applied to whole proximal femora of different mammalian species to investigate the algorithm’s potential to predict actual joint loads (Christen et al. 2015).

Although the preliminary results of micro-FE-based joint load predictions are promising (Christen et al. 2015), parameter sensitivity and validity of the bone load estimation algorithm remain to be investigated in detail. While load predictions have shown to be robust with respect to CT image resolution as long as the voxel size remains below $80 \,\upmu \hbox {m}$ (Christen et al. 2016), the influence of other parameters associated with high uncertainty such as bone material properties (Lucchinetti et al. 2000), remodelling equilibrium stimulus (Mullender and Huiskes 1995), or pressure distribution at the joint (Fischer et al. 1995, 1999; Bona et al. 2006) has not yet been assessed. Furthermore, the validation of joint load predictions has so far been limited to the comparison of the peak load magnitude of a single human proximal femur (Christen et al. 2015) to hip joint loads measured in vivo (Bergmann et al. 2001).

Building on previous work, the goals of this study were (1) to conduct a systematic investigation of the parameter sensitivity of micro-FE-based joint load predictions on one human proximal femur and (2) to assess the plausibility of the results by comparing the predicted joint load vectors (i.e. magnitude and direction) of ten proximal femora to hip joint loads measured in vivo (Bergmann et al. 2016).

## Materials and methods

### Study outline

A graphical outline of the study is presented in Fig. 1. Joint load predictions following the algorithm of Christen et al. (2012) were performed using micro-FE models generated from high-resolution computed tomography (CT) scans of human proximal femora (see Sect. 2.2). One proximal femur was used to investigate parameter sensitivity by predicting peak and mean joint load vectors and comparing them between a reference model and several parameter-varied models (Fig. 1, left, see Sect. 2.3). The plausibility of the algorithm was assessed by comparing predicted peak joint load vectors and load ranges of ten femora with in vivo hip joint loads of ten subjects reported by Bergmann et al. (2016) (Fig. 1, right, see Sect. 2.4).

### Micro-FE-based joint load prediction

#### Image processing

**Fig. 2:**
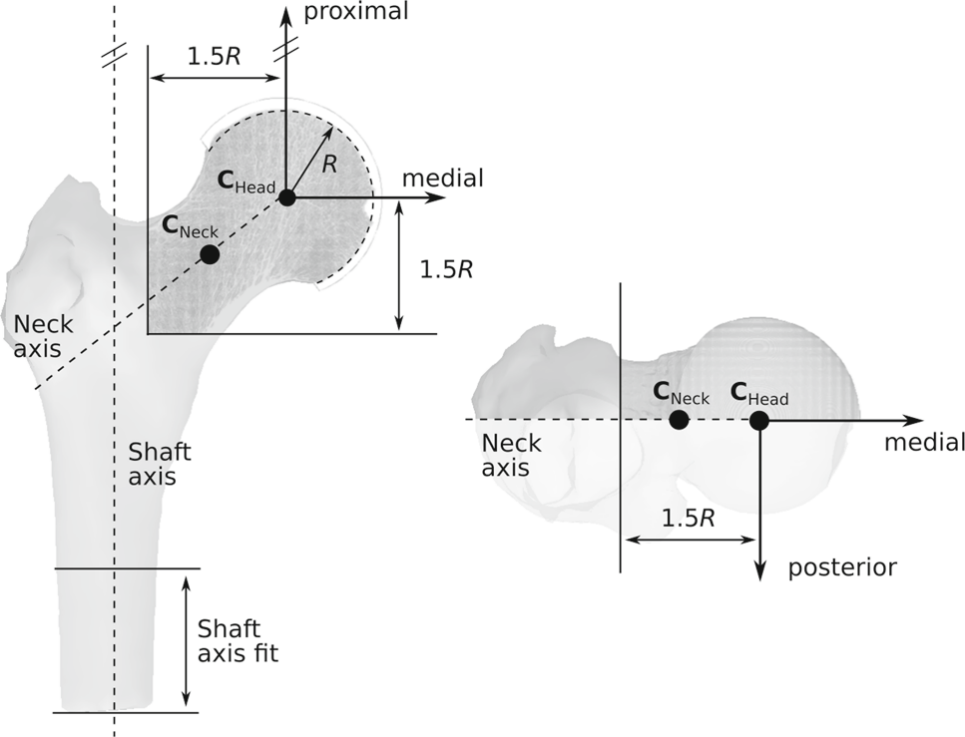
Definition of the specimen-specific coordinate systems and bounding boxes in anterior (left) and superior (right) views. *R* is the specimen-specific femoral head radius, $\mathbf {C}_{\mathrm {Neck}}$ is the midpoint of the femoral neck with smallest cross section, and $\mathbf {C}_{\mathrm {Head}}$ is the centre of the femoral head

Ten human proximal femora (age: $81.9\pm 8.7 \hbox { years}$, left/right: 7/3) were collected under permission of the german law “Gesetz über das Leichen-, Bestattungs- und Friedhofswesen des Landes Schleswig-Holstein, Abschnitt II, 9 (Leichenöffnung, anatomisch)” from 04.02.2005 at the Anatomy Institute of the Lübeck University. They were cut to approximately 160 mm length and scanned with an isotropic resolution of 30.3 $\upmu$m using a high-resolution peripheral quantitative CT scanner (XtremeCT2, Scanco Medical AG, Brüttisellen, Switzerland, energy: 68 kVp, intensity: $1470~\upmu \hbox {A}$). The three-dimensional (3D) images were resampled by a factor of two (voxel size $60.6 \,\upmu \hbox {m}$) to reduce computational effort without compromising the results of the load prediction (Christen et al. 2016). A coordinate system was defined in each femur which was consistent with the “implant coordinate system” used to measure joint loads with instrumented prostheses (Bergmann et al. 2016) (Fig. 2). The origin of the coordinate system was located in the centre of the femoral head, defined as the centre of the best-fitting sphere. The vertical axis of this coordinate system was parallel to the shaft axis of the bone, which was defined by fitting a straight line to the shaft centroids of a 40-mm-long section at the distal end of the bone. The anterior–posterior axis was defined as perpendicular to both the vertical and the neck axis. The neck axis was determined by the line connecting the femoral head centre and the midpoint of the femoral neck where the cross-sectional area is smallest (Väänänen et al. 2015). Finally, the medio-lateral axis was defined as perpendicular to both the vertical and the anterior–posterior axes.

CT scans of all femora were rotated into the new coordinate system and cropped by bounding boxes extending 1.5 times the femoral head radius in both lateral and distal directions. This size was chosen to reduce computational effort while still covering regions of dominant stresses and strains resulting from hip joint loading (Cristofolini et al. 2007). All images were filtered using a Gaussian filter (support: 2 voxels, $\sigma =1.6$) to reduce image noise and segmented using a fixed threshold with a constant value for all specimens (greyvalue: 3000). The threshold was chosen manually after visual inspection of both the image histograms and segmentation results. Finally, a spherical layer of elastic material mimicking cartilage was added to all specimens to facilitate the load application on the FE models (see Sect. 2.2.2). The selected thickness of this layer (2.2 mm) was kept as small as possible but large enough to ensure that no bone material penetrated through its surface.

A representative specimen after image processing is displayed in Fig. 2. All image processing steps were performed using medtool 4.0 (Dr. Pahr Ingenieurs, Pfaffstätten, Austria) and additional custom Python scripts.

#### FE models

**Fig. 3:**
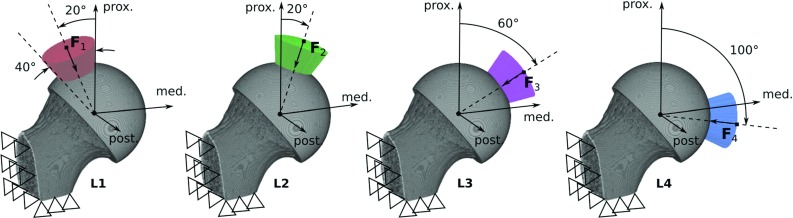
Micro-FE models with different sets of boundary conditions representing the four unit load cases (L1 to L4) with resultant force vectors $\mathbf {F}_1$ to $\mathbf {F}_4$ used in the joint load prediction algorithm. All resultant force vectors were within a single plane (frontal plane). Coloured regions indicate the size of the load area and the direction of nodal force vectors. Open triangles indicate constrained surfaces

To apply the load prediction algorithm, mechanical stimuli need to be evaluated from FE models representing different unit load cases. For this purpose, voxel-based FE models (element size $60.6~\upmu \hbox {m}$) with four different sets of boundary conditions were generated (Fig. 3). Nodes at the lateral and distal boundary were always fully constrained, and distributed loads were applied for load cases L1 to L4 with resultant forces inclined by $-20^{\circ }$, $20^{\circ }$, $60^{\circ }$, and $100^{\circ }$, respectively. The number of load cases was limited to four to avoid problems associated with overlapping load areas (for further explanation, see results and discussion section of the parameter sensitivity analysis in Sects. 3.2 and 4) while still covering a meaningful range of force directions in the frontal plane. It was assumed that the load distribution is uniform and that all nodal force vectors act normal to the joint surface. The shape of the load area was circular to follow the idealized assumptions of a sphere-to-cup contact. The size of each load area was defined by the intersection of the spherical joint surface and a cone with an opening angle of $40^{\circ }$, resulting in an area of $224.24\pm 23.44 \hbox { mm}^2$ for all specimens. The resultant force magnitude of each load case was set to 1000  N.

**Fig. 4:**
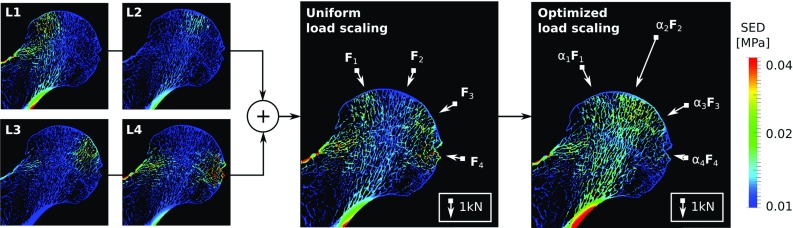
Graphical explanation of the load prediction algorithm following Christen et al. (2012) using one representative specimen of this study. SED distributions from four unit load cases L1 to L4 are superimposed and optimally scaled by factors $\alpha _1$ to $\alpha _4$ such that the difference to a remodelling equilibrium SED (typically 0.02 MPa) is minimized. White arrows indicate the scaled resultant force associated with each of the four unit load cases. The cartilage layer is not displayed

Linear elastic, isotropic material was assumed for both the bone material and the cartilage layer. Material properties were defined following the study of Christen et al. (2012) where load predictions were in good agreement with in vivo loads in whole mice vertebrae: Elastic moduli were set to 10 GPa and 10 MPa for bone and cartilage, respectively, and the Poisson’s ratios were assumed to be 0.3 for both. The final micro-FE models had $473.0\pm 69.2$ million degrees of freedom and were solved using the parallel octree solver ParOSol (Flaig 2011).

#### Joint load prediction algorithm

The joint load prediction was performed using the algorithm presented by Christen et al. (2012) (see Fig. 4). The underlying assumption of the algorithm is that the observed bone structure is the result of a simple remodelling law: Bone is either added or resorbed unless the local mechanical stimulus equals a certain remodelling equilibrium stimulus. Consequently, the most probable bone loading history is the one most closely leading to remodelling equilibrium within the whole bone.

The loading history is represented by a finite number of *n* unit load cases, which are assumed to act with a magnitude $\alpha _i$ for $m_i$ load cycles within an observed timeframe. The local mechanical stimulus $U(\mathbf {x})$ at location $\mathbf {x}$ within the bone is then computed by summarizing the strain energy densities (SED) $U_i(\mathbf {x})$ resulting from unit load cases 1 to *n*, weighed by their relative number of load cycles $m_i/m_{\mathrm{tot}}$ and magnitude $\alpha _i$:

1 $$\begin{aligned} U(\mathbf {x}) = \sum \limits _{i=1}^n \frac{m_i}{m_{\mathrm {tot}}} \cdot \alpha ^2_i \cdot U_i(\mathbf {x}) \end{aligned}$$

Introducing the combined scaling factor $s_i = \alpha _i^2 \cdot m_i/m_{\mathrm{tot}}$ then allows to find the most probable loading history by solving a simple optimization problem which minimizes the difference between the local mechanical stimulus $U(\mathbf {x})$ and the remodelling equilibrium stimulus $\tilde{U}$ at all locations $\mathbf {x}$ within the bone:

2 $$\begin{aligned} \begin{aligned}&\underset{s_i}{\text {minimize}}&\sum _{\mathbf {x}\in \mathcal {X}} \left[ \tilde{U} - \left( \sum \limits _{i=1}^n s_i \cdot U_i(\mathbf {x}) \right) \right] ^2 \end{aligned} \end{aligned}$$

Solving Eq. (2) for the optimal scaling factors $s_i$ and assuming a constant number of load cycles for all *n* unit load cases (Christen et al. 2012), the load magnitude $\alpha _i$ can be computed as follows:

3 $$\begin{aligned} \alpha _i = \sqrt{ n \cdot s_i } \end{aligned}$$

**Table 1:** Overview of the reference and varied parameters used in the parameter sensitivity analysis

Parameter	Icon	Reference value	Variation 1	Variation 2
Image segmentation threshold	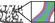	3000	3300	2700
Load area	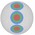	$215.1 \hbox { mm}^2$	$121.5 \hbox { mm}^2$	$54.2 \hbox { mm}^2$
Nodal force distribution/alignment	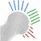	Uniform/surface normal vectors	Uniform/parallel vectors	Ellipsoidal/parallel vectors
Bone elastic modulus	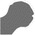	10 GPa	5 GPa	20 GPa
Cartilage elastic modulus	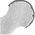	10 MPa	100 MPa	1000 MPa
Number of unit loads	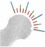	4	7	13
Unit load location	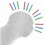	$0^{\circ }$	$+\,10^{\circ }$ rotation	$-\,10^{\circ }$ rotation
Region of interest	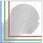	Full model	5 mm reduction	10 mm reduction
Equilibrium stimulus		0.02 MPa	0.01 MPa	0.04 MPa

The colours blue, green, and orange of the icons refer to the reference value, variation 1, and variation 2, respectively

In this study, the optimization problem presented in Eq. 2 was solved in Python using the non-negative least squares algorithm of SciPy (Jones et al. 2001). The remodelling equilibrium stimulus $\tilde{U}$ was set to 0.02 MPa as estimated by Mullender and Huiskes (1995) and used in previous studies (Christen et al. 2012, 2015, 2016). Finally, joint load vectors were computed by multiplying the resultant force $\mathbf {F}_i$ of each unit load case *i* with the corresponding load magnitude scaling factor $\alpha _i$. A graphical overview of this procedure is shown in Fig. 4.

The quality of the load prediction algorithm was quantified by comparing tissue loading homogeneity before and after optimization of the unit load scaling factors. Tissue loading inhomogeneity was quantified by the coefficient of variation (CoV) of the distribution of the mechanical stimuli $U(\mathbf {x})$ (Christen et al. 2012) (see Eq. 1). A Wilcoxon signed-rank test was applied to verify whether the CoV was significantly reduced after optimization.

### Parameter sensitivity of the predictions

Parameter sensitivity of the joint load predictions was analysed by comparing the results of one specimen with a set of reference parameters (as described in Sect. 2.2) to those obtained after variation. In total, nine parameters with two variations each were investigated as listed in Table 1 and explained below. Variations of each parameter were tested separately, while keeping all other parameters constant (i.e. the reference value).

#### Image processing parameters

Previous studies have shown that image segmentation thresholds can affect morphometric measurements and mechanical properties evaluated with micro-CT and -FE methods (Hara et al. 2002; Chevalier et al. 2007). Thus, the influence of image segmentation was investigated by increasing (“variation 1”) or reducing (“variation 2”) the threshold greyvalue separating bone from the background. The sensitivity of load predictions to image resolution was addressed in a previous study (Christen et al. 2016) and therefore is not tested here.

#### FE model parameters

Variations in the boundary conditions were investigated by changing the load area size as well as the distribution and alignment of nodal force vectors. In contrast to the reference configuration, nodal force vectors were considered to be parallel and uniformly distributed (“variation 1”) or distributed following an ellipsoidal (Hertzian) pressure distribution (“variation 2”) (Table 1). In all cases, the resultant force magnitude was set to 1000 N. Material properties of bone were varied to account for the large range of elastic moduli reported in the literature ranging from 1 to 25 GPa (Lucchinetti et al. 2000; Zysset et al. 1999). Additionally, the cartilage layer material was varied from soft (“reference value”) to very stiff (“variation 2”) (Table 1).

#### Joint load prediction algorithm parameters

The algorithms robustness was investigated by increasing the number of unit loads, shifting the location where unit loads were applied, reducing the size of the region of interest (ROI) of the SEDs included in the optimization, and varying the remodelling equilibrium stimulus, as shown in Table 1. The number of unit loads was increased by generating and solving additional FE models with loading applied in regular intervals between $-\,20^{\circ }$ and $100^{\circ }$ inclination. Unit load location was varied by solving additional FE models with unit load resultant forces rotated $\pm \,10^{\circ }$ around the anterior–posterior axis (for a definition of the anatomical axes see Figs. 2 and 3). The ROI size was reduced by 5 and 10 mm at the lateral and distal boundary with respect to the original model dimensions. Finally, the remodelling equilibrium stimulus was varied from 0.01 to 0.04 MPa as the commonly used value of 0.02 MPa is known to be only a rough estimation (Mullender and Huiskes 1995).

#### Output variables

Peak and mean vectors of the joint load predictions were evaluated for the parameter sensitivity analysis. Mean vectors were defined as the sum of the scaled resultant force vectors of each load case divided by the number of load cases. Mean vectors were used as an output variable to quantify differences in the load predictions, irrespective of changes in the number and/or location of unit loads. Additionally, unit load case-specific force magnitudes were compared qualitatively.

### Plausibility of the predictions

The plausibility of the joint load prediction results was assessed by comparing load prediction results from all ten femora (as described in Sect. 2.2 with reference parameters as shown in Table 1) with the in vivo resultant hip joint forces presented by Bergmann et al. (2016) (accessed from www.orthoload.com, dataset “Standard Loads Hip Joint”). Data selection and processing are described in the following sections.

**Table 2:** Load magnitude scaling factors $\alpha _i$ after optimization and the coefficient of variation (CoV) quantifying tissue loading inhomogeneity

Specimen	${\alpha _1}$ (−)	${\alpha _2}$ (−)	${\alpha _3}$ (−)	${\alpha _4}$ (−)	$\hbox {CoV}_\mathrm {init}$ (%)	$\hbox {CoV}_\mathrm {opt}$ (%)
1	1.25	3.32	1.39	0.65	203.83	137.55
2	1.02	2.47	0.12	0.97	403.04	146.89
3	0.97	3.92	0.22	0.08	1515.66	135.75
4	0.45	3.61	1.09	0.53	226.13	135.88
5	1.21	3.60	0.23	0.55	441.30	128.81
6	1.34	3.86	0.93	0.72	213.06	132.03
7	0.50	4.18	0.28	0.97	430.77	155.68
8	1.12	2.35	0.96	0.20	425.26	129.13
9	0.14	3.25	0.25	0.29	629.30	151.00
10	0.79	3.17	1.18	0.83	188.30	140.20
Mean	0.88	3.37	0.66	0.58	467.67	139.29
SD	0.40	0.60	0.49	0.31	394.86	9.17

The CoV was reduced significantly ($p<0.05$) between uniformly ($\hbox {CoV}_\mathrm {init}$) and optimally ($\hbox {CoV}_\mathrm {opt}$) scaled unit loads. *SD* standard deviation

#### OrthoLoad data selection and processing

In the study by Bergmann et al. (2016), hip joint forces were reported for ten human subjects (age: $56.9\pm 5.5$ years, weight: $88.7\pm 13.1$ kg) during the most common activities of daily living (Morlock et al. 2001). Load data from the following activities were used for this study: walking at a self-determined speed, stair climb and descend without handrail, standing up, sitting down, and one legged stance. The measured forces were transformed into the “implant coordinate system” as described by Bergmann et al. (2016) for comparison with the micro-FE-based predictions. Subject-specific peak loads were defined as the forces with largest magnitude throughout the full loading cycles of all activities. The range of force directions was evaluated by computing the maximum and minimum inclination angle with respect to the vertical axis in the frontal plane based on all resultant forces (considering full load cycles, all subjects, and all activities).

#### Output variables

Subject-specific in vivo peak loads were compared to the peak load vectors obtained from the micro-FE-based load prediction. Peak load directions were quantitatively compared based on the angles of the force vectors with respect to the vertical axis in the frontal plane. Additionally, the range of force directions predicted by the FE models was compared to the full range of force directions measured in vivo.

## Results

### Joint load prediction algorithm results

Load magnitude scaling factors $\alpha _i$ for all ten proximal femoral specimens are presented in Table 2. Tissue loading inhomogeneity (CoV) was significantly reduced ($p=0.005$) using the optimized load scaling factors. Furthermore, the CoV standard deviation was also considerably reduced after optimization.

**Fig. 5:**
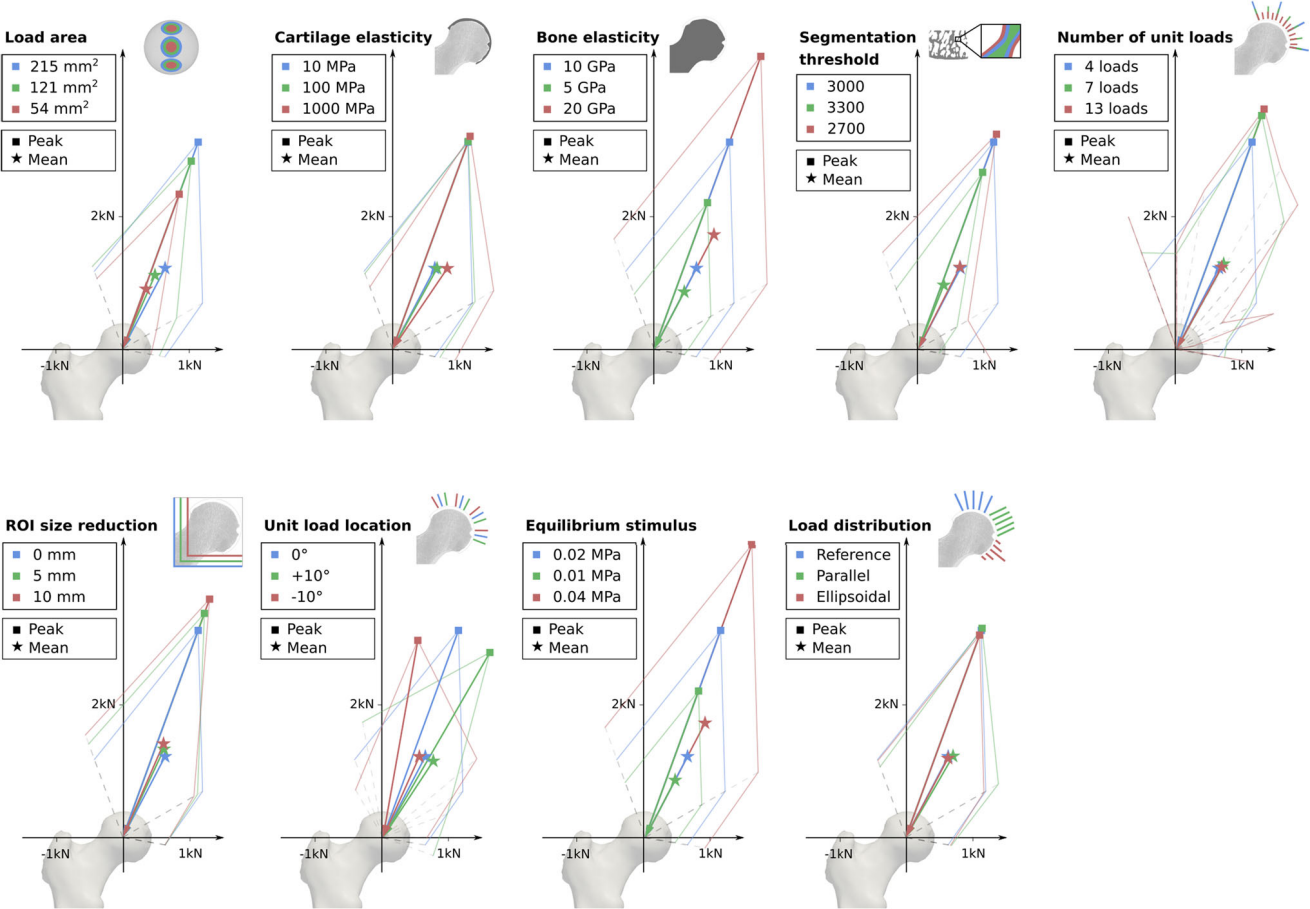
Results of the parameter sensitivity study. Load predictions of one specimen with reference parameters (blue) are compared to the results after two parameter variations (green, orange). Squares and stars indicate peak and mean joint load vectors, respectively. The faint lines connect the predicted load magnitude for each unit load case

### Parameter sensitivity of the predictions

Figure 5 shows the joint load prediction results after varying nine different parameters in one specimen. In general, a single peak of the joint loads was predicted at roughly $20^{\circ }$ inclination with respect to the vertical axis of the femur and load magnitudes decreased towards the boundaries of the articular surface. This pattern was robust against variations of parameters except for changes in the number of unit loads. More than four unit loads caused fluctuations in the predictions without further considerably reducing the remaining tissue loading inhomogeneity ($\mathrm{CoV}=137.5$ and 134.5% for 4 and 13 unit loads, respectively).

The predicted peak joint load vector in the reference specimen was inclined by $20^{\circ }$ and had a magnitude of 3316 N. Directions of peak joint load vectors were robust against all parameter variations except for changes in the unit load location (range of differences: $-\,10$ to $+\,10^{\circ }$). In contrast, the magnitudes of the predicted peak loads were more sensitive to variation in parameters, particularly changes to the load area size, segmentation threshold, bone elastic modulus, and equilibrium stimulus (range of differences: $-\,971.2$ to $+\,1373.5$ N). Changes to cartilage elasticity, number of unit loads, ROI size, unit load location and load distribution had a limited effect on the predicted peak joint load magnitude (range of differences: $-\,484.6$ to 529.1 N).

The mean joint load vector in the reference specimen was inclined by $27.6^{\circ }$ with a magnitude of 1373.3 N. Directions of mean joint load vectors were even less sensitive to parameter variations (range of differences: $-\,6.5$ to $+\,6.6^{\circ }$) than the direction of peak joint load. In contrast, magnitudes of mean forces were sensitive to changes in the parameters (range of differences: $-\,404.7$ to $+\,568.9$ N).

### Plausibility of the predictions

**Fig. 6:**
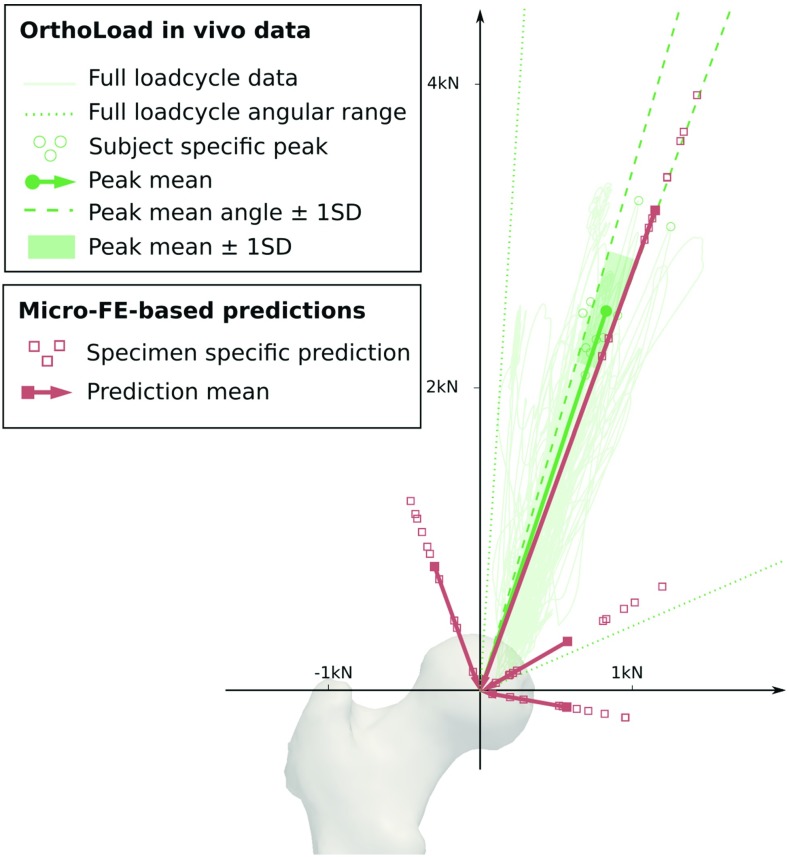
Comparison of micro-FE based hip joint load predictions (red) with in vivo data (green) measured by instrumented prostheses [from Bergmann et al. (2016)] in the frontal plane. *SD* standard deviation

Figure 6 shows the results of the load prediction for all ten femora (red) and the in vivo hip joint loads of ten subjects (green) from Bergmann et al. (2016) in the frontal plane. The micro-FE predicted peak joint loads of all femora were in good agreement with the subject-specific peak resultant hip joint forces measured in vivo. Predicted peak load directions of $20^{\circ }$ were within one standard deviation of the in vivo data ($18.2\pm 2.0^{\circ }$). However, predicted magnitudes of $3372.2\pm 597.9$ N exceeded those measured in vivo ($2707.6\, \pm \, 443.3$ N).

The in vivo range of resultant force vectors was confined to inclinations of $3.7^{\circ }$ to $66.6^{\circ }$ with respect to the vertical axis of the femur. In contrast, resultant forces predicted with the micro-FE models ranged from $-\,20^{\circ }$ to $100^{\circ }$ (i.e. all scaling factors were nonzero).

## Discussion

The goal of this study was to investigate the parameter sensitivity and plausibility of micro-FE-based prediction of joint loads. A comparison with in vivo loading data of the hip joint showed that peak load predictions were plausible in terms of both load magnitude and direction. However, particularly the *magnitudes* of the load predictions have to be interpreted with caution considering their sensitivity to parameters associated with high uncertainty such as bone material properties and remodelling equilibrium stimulus.

The results of the parameter sensitivity analysis highlight many important factors to be considered when utilizing and interpreting micro-FE-based load prediction following Christen et al. (2012). First, the number of applied unit load cases was found to highly influence individual load scaling factors without considerably affecting the remaining tissue loading homogeneity. This indicates the non-uniqueness of the solution when the loading areas of unit loads overlap and could also explain the large fluctuations of load scaling factors observed in earlier studies (Christen et al. 2015). Second, the predicted load magnitudes were considerably affected by parameters with high uncertainty and/or variability such as bone material properties, remodelling equilibrium stimulus, and area of load application. The equilibrium stimulus in particular is still not accurately defined and might lie in a range as large as 0.001–0.068 MPa (Mullender and Huiskes 1995; Lucchinetti et al. 2000). This uncertainty dramatically affects the predicted load magnitudes, as a variation of the equilibrium stimulus from 0.01 to just 0.04 MPa in this study already elicited a 2000 N change in the predicted load magnitudes. Although the load *magnitude* might be biased by the selection of bone material properties and equilibrium stimulus, predicted *directions* of both peak and mean vectors were less sensitive to variations in these parameter. Also, other potential sources of influence such as the cartilage elasticity, ROI of SEDs used in the optimization, and load distribution were shown to have a limited effect on both the load directions and magnitudes. Overall, our results suggest that micro-FE-based load predictions are potentially robust enough to compare dominant joint loads between different bones using the same set of parameters, and that predicted directions of loads are robust even when parameters vary. However, absolute values of load magnitudes should be interpreted with caution until validated parameters are available.

The comparison of joint load predictions of the full sample with the in vivo hip joint load data (Bergmann et al. 2016) showed that predicted peak loads were plausible both in terms of their direction and magnitude. However, large joint loads ($>500$ N) were also predicted in directions outside the range of in vivo values. This might be explained by two factors: First, the joint load prediction presumes that bone structure is solely the result of a mechanical stimulus. In reality, bone architecture is also influenced by other factors such as genetics, calcium homeostasis, and hormone levels (Si and Rodan 2003; Abel and Macho 2011; Burr 2002; Rodan 1991). Second, the large range of joint loads might be an artefact resulting from the assumption of a simple uniform pressure distribution. The actual pressure distribution might be horse-shoe-shaped due to joint incongruity (Afoke et al. 1987; Eisenhart et al. 1999) and trigger bone formation also in locations close to the boundary, while the resultant force directions would still be in line with the in vivo loading data (Fischer et al. 1999; Bona et al. 2006).

Several limitations of this study remain to be mentioned. First, micro-FE predictions were performed on specimens obtained from elderly donors (age $80.5\pm 7.6$ years). Changes of bone structure and particularly bone density with age are well-documented (Macho et al. 2005; Berger et al. 2008) and might influence the results. However, it was expected that the bones in this study sample were still adapted to loads from activities with moderate intensity such as level walking or stair climbing. Second, loading conditions were highly simplified. The assumption of circular load areas with static size and uniform load distributions are likely not physiologically realistic (Eisenhart et al. 1999; Bona et al. 2003; Fischer et al. 1999). More complex shapes of the load areas and pressure distributions or even inclusion of bone-to-bone contact (Bona et al. 2006) might improve the results but exceeded the scope of this study. Third, only four unit load cases were used to compare micro-FE-based predictions to in vivo joint loads. The number of unit loads and size of the loading areas were chosen to allow identifying the plausibility of peak loads as accurate as possible without introducing load scaling fluctuations due to overlapping loading areas (as described above and shown in Fig. 5). Additional load cases at the posterior and anterior side of the joint could have been added without overlap, but were avoided to reduce computational effort. Fourth, the in vivo data used in this study were collected in patients with instrumented prostheses. The hip replacement itself might lead to differences of the joint loads when compared to healthy subjects (Stansfield and Nicol 2002; Wesseling et al. 2016). Finally, the load estimation algorithm of Christen et al. (2012) is based on a highly simplified bone remodelling theory. Although there is evidence that bone formation and resorption are generally related to local mechanical loading (Christen et al. 2014), many other aspects of remodelling such as the influence of load cycle number and load amplitude (Kivell 2016; Rubin et al. 2002; Umemura et al. 1997) or the existence of a lazy zone (Frost 1987; Christen et al. 2014) are still disputed and require further investigation.

Overall, the results of this study suggest that micro-FE-based joint load predictions deliver plausible estimates of the most dominant loading experienced by a given bone structure. Load predictions are potentially robust enough to perform inter-subject or inter-species comparisons of joint loads, but absolute values should be interpreted with caution considering both parameter sensitivity and many limitations inherent to the load estimation algorithm.
